# Physical therapy intervention for breast symptoms in lactating women: a randomized controlled trial

**DOI:** 10.1186/s12884-023-06114-2

**Published:** 2023-11-14

**Authors:** Kuan-Yin Lin, Wei Shao, Yi-Ju Tsai, Jeng-Feng Yang, Meng-Hsing Wu

**Affiliations:** 1 Department of Physical Therapy, College of Medicine, National Cheng Kung University, Tainan, Taiwan; 2 School and Graduate Institute of Physical Therapy, College of Medicine, National Taiwan University, Taipei, Taiwan; 3 Institute of Allied Health Sciences, College of Medicine, National Cheng Kung University, Tainan, Taiwan; 4 Physical Therapy Center, National Cheng Kung University Hospital, Tainan, Taiwan; 5 Department of Obstetrics and Gynecology, College of Medicine, National Cheng Kung University, Tainan, Taiwan; 6 Department of Obstetrics and Gynecology, College of Medicine, National Cheng Kung University Hospital, National Cheng Kung University, Tainan, Taiwan

**Keywords:** Physical therapy, Ultrasound therapy, Massage, Breastfeeding, Randomized controlled trial

## Abstract

**Background:**

Therapeutic ultrasound, education, and massage are the most common physical therapy interventions provided to mothers with breast symptoms. However, there is insufficient evidence on the effectiveness of the combination of these interventions. This study aimed to explore the effects of the combination of therapeutic ultrasound, education, and massage on breast symptoms in lactating women.

**Methods:**

This study was a single-blind randomized controlled trial. Postpartum lactating women aged from 21 to 45 with breast symptoms were recruited and randomly allocated to one of three groups (ultrasound group, sham group, and usual care group). The severity of breast symptoms (pain, redness, lump, general malaise), breast engorgement, breast hardness, body temperature, breast temperature, and milk volume were assessed at baseline (T1), immediately post-intervention (T2), and at 3 months following baseline (T3).

**Results:**

A total of 37 participants were included in the study (ultrasound group n = 12; sham group n = 12; usual care n = 13). The severity of breast symptoms (i.e., pain, lump, and general malaise) as well as breast engorgement, were significantly improved in the ultrasound group at T2 when compared to T1, and these improvements were sustained at T3. The severity of breast engorgement was significantly lower in the ultrasound group when compared to the usual care group at T2. However, no statistically significant differences were found between the ultrasound and sham groups for all outcomes at any assessment time points.

**Conclusions:**

Physical therapy interventions may be beneficial in relieving breast symptoms in lactating women. Larger randomized controlled trials are needed to confirm the findings of this study.

**Trial registration:**

ClinicalTrials.gov (NCT04569136); Date of registration: 29/09/2020.

## Background

Breast pain, tenderness, redness, engorgement, fever, malaise, chills, lethargy, sweating, headache, nipple damage, and a hot spot on the breast [1, 2] are common complaints in women who breastfeed. Despite the high prevalence of breast symptoms in lactating women during the first 12 weeks after childbirth [3], some breast symptoms can occur or persist throughout the entire period of lactation, which may last up to six months [4, 5]. If these breast symptoms [6]are not managed and treated during early postpartum clinic visits, the discomfort of the lactating breast may lead to various breast disorders, such as blocked milk ducts, mastitis, and abscess [6–8], potentially resulting in early cessation of breastfeeding [4, 9].

Therapeutic ultrasound (91%); education and advice regarding eliminating duct obstruction/trauma, feeding, lifestyle changes, thermal/cryo therapy, and alternative interventions (83%); and massage (54%) have been reported as the three most common interventions provided by the physical therapists for breast symptoms in lactating women [10–12]. However, postpartum women are often unaware of physical therapy treatment options for breast symptoms, with some women having only been prescribed antibiotics and/or encouraged by the doctor to use active expression of human-milk to relieve the symptoms [13]. The majority of women felt that care for breastfeeding women with breast symptoms could be improved and were desperate for help from healthcare professionals [13].

A Cochrane review identified 17 different interventions for breast engorgement in breastfeeding women and concluded that there is insufficient evidence on the effectiveness of any intervention for breast engorgement to justify widespread implementation [11]. Furthermore, the evidence on the effects of ultrasound, education, and massage delivered in combination [14, 15] is inconsistent due to various intervention designs. Therefore, more randomized controlled trials (RCTs) are needed to draw evidence-based conclusions regarding the effects of the combination of physical therapy interventions (i.e., therapeutic ultrasound, education, and massage) for breast symptoms during lactation.

This study aimed to explore the effects of the combination of therapeutic ultrasound, education, and massage on breast symptoms in lactating women and to compare them to patients receiving sham ultrasound treatment and usual obstetric care. We hypothesized that the outcomes of interest would improve following the physical therapy interventions, and the ultrasound group would show a greater improvement in the outcomes compared to the sham and usual care groups.

## Methods

### Research design

This is a prospective, assessor-blinded single-center RCT. This study is reported according to the CONSORT guidelines [16]. Ethics approval was obtained from National Cheng Kung University Hospital (Institutional Review Board [IRB] No: --/ B-BR-109-028). This study was registered with ClinicalTrials.gov, number NCT04569136, before commencement.

### Setting and relevant context

Participants were recruited through the Obstetrics and Gynecology Clinic of National Cheng Kung University Hospital, postpartum care centers, websites, and the community. Approximately 1,500 women give birth annually at the National Cheng Kung University Hospital. The research was conducted in the postnatal ward of the National Cheng Kung University Hospital, and the postpartum care centers; the participating centers provided lactation support from International Board Certified Lactation Consultants and Chinese Certified Lactation Assistants.

### Sample

Patients were eligible for inclusion if they had given birth at the National Cheng Kung University Hospital, were breastfeeding, aged 21–45, had breast symptoms, and had sufficient Chinese/Mandarin language skills to participate. Patients were excluded if they had a history of breast reduction or augmentation, an abscess, severe physical/psychiatric impairments, or any malignancies.

Sample size calculation was based on data from McLachlan 1991 using changes in pain scores following ultrasound treatment for breast engorgement [15]. These data indicated an effect size of 1.69 for the change in pain measured using the visual analog scales from baseline to post-final treatment [15]. Setting power at 80% and alpha at 5%, a sample size of five participants in the ultrasound group was required. Furthermore, based on the differences in pain scores in the study by Habibu and Hanif [17], we expected an effect size of 2.78 for the experimental group (non-thermal ultrasound and conventional treatment) as compared to the control group (conventional treatment). With an alpha of 0.05 (two-sided), we would need four participants in each group to achieve a power of 0.80. Allowing 20% missing data, we factored in over-recruitment by 20%. We estimated that an overall sample of 11 participants per group (total n = 33) would be sufficient to detect a significant difference in the primary outcome of breast pain within the ultrasound group and between the three groups after intervention.

### Measurement

Outcome variables were collected at baseline (T1), immediately post-intervention (T2), and three months after the intervention period (T3). Socio-demographic and health variables were measured using a questionnaire at T1.

#### Primary outcome

Breast pain in the past 24 h was assessed using the numerical rating scale (NRS) [18]. Participants were asked to rate breast pain from 0 to 10 (11-point scale), with 0 indicating no pain and 10 indicating the worst possible pain [18]. The NRS is a valid and reliable scale to measure pain intensity and is sensitive to detect changes after physical therapy treatments [18].

#### Secondary outcomes

The degree of breast engorgement of the left and right breasts was assessed using a six-point engorgement scale developed by Hill and Humenick [19]. Higher scores indicate more severe breast engorgement (1 = soft, no change, 2 = slight change, 3 = firm, non-tender, 4 = firm, beginning tenderness, 5 = firm, tender, 6 = very firm and very tender) [19]. The Chinese version has satisfactory internal consistency (Cronbach’s alpha 0.74) [20].

Breastfeeding self-efficacy was evaluated using the Breastfeeding Self-Efficacy Scale – Short Form (BSES-SF). The BSES-SF is a validated and reliable patient-reported outcome measure consisting of 14 items [21]. Each item is scored between 1 and 5 points with 1 indicating ‘not at all confident’ and 5 indicating ‘always confident’. Total scores range from 14 to 70, with higher scores indicating higher breastfeeding self-efficacy [22].

The assessor used a portable durometer (NEUTONE TDM-Z2 (CL); TRY-ALL corp.) to measure the hardness of the left and right breasts. The participant was in a supine position, and the durometer was placed at 3 cm from both nipples in the 10 o’clock and 2 o’clock positions [23, 24]. The spring-loaded presser was pressed downwards until the trigger switched on [25]. Each position was measured three times and the mean value was obtained. The durometer is a reliable and useful device for objective quantitative evaluation of soft tissue stiffness [25] and has been used in a breast study [26].

Core body and breast temperatures were measured using a non-contact infrared thermometer on the central part of the forehead and in the area 3 cm from both nipples in the 10 o’clock and 2 o’clock positions, respectively [23]. The temperature of each position was measured three times and the mean value was recorded. The non-contact infrared thermometer is a reliable, and accurate means for measurement of temperature [27].

As massage may affect the human-milk volume in postpartum women [28], the volume of the human-milk was collected by an electric breast pump for 15 min. Human-milk volume was recorded by weighing the collecting bottle on an electronic digital scale to the nearest 0.1 g before and after each expression [29].

Satisfaction and acceptability of the intervention program were assessed immediately post-intervention using a short questionnaire with a 7-point Likert scale response to questions of ‘How acceptable did you find the physical therapy interventions used in this study?‘ and ‘How would you rate your overall satisfaction with the physical therapy program?‘

Adverse events were recorded and reported as per National Cheng Kung University Hospital IRB guidelines on reporting adverse events and serious adverse events, with the absence of adverse or serious adverse events related to the physical therapy intervention used indicating the safety of the trial protocol.

### Data collection

The study was conducted from May 2021 to April 2023. Treating medical staff identified potentially eligible patients with breast symptoms, and provided them with a summary information sheet about the study. Eligible patients who agreed to find out more information about the study were seen after their medical appointment by the on-site researcher who explained the study, went through the participant information and consent form, and invited them to participate. Participants recruited from the postpartum care centers, websites, and communities through the flyers posted were self-identified as having breast symptoms and contacted the research team. After the eligibility was confirmed by the researcher and the consent form was signed, the first assessment was arranged.

Consented participants were randomized by an independent, off-site, computer-generated random number sequence into three groups with a 1:1:1 ratio: ‘ultrasound’ group, ‘sham’ group, and ‘usual care’ group. The research team accessed the allocation for each enrolled participant via the web-based application at the time of allocation and informed the participant and the treating physical therapist of the allocated group. Participants and the physical therapists providing the intervention program were not blinded to the intervention allocation. Researchers performing assessments were not involved in the delivery of the intervention and were blinded to the group allocation of participants.

### Intervention

#### Ultrasound group

Participants allocated to the ‘ultrasound’ arm of the trial participated in four treatment sessions with the physical therapist over one week; each session lasted up to one hour [30]. The focus of the initial appointment was educating the participant about common breast symptoms and self-management strategies, treating with therapeutic ultrasound, and administering and teaching breast massage.

The education session including education about common breast symptoms, feeding techniques, lifestyle changes, thermo/cryo therapy, and demonstration of breast self-massage was approximately 20 min. A five-minute breast self-massage was taught, and participants were asked to apply light pressure on the breast [31] and demonstrate it to the physical therapist. Participants were encouraged to perform the massage at least three times a day before hand expression. The massage started with wiping the breast with a hot towel and then applying lubricants (creams or oils) to the area to be treated. The steps of massage [32] included [1] superficial stroking from the areola toward the armpit (repeated three times), [2] palmar kneading the breast in alternating clockwise and counterclockwise motions (repeated three times), [3] knuckle kneading two sides of the breast toward the nipple (repeated three times), [4] tapping around the breast region with the fingertips in a circular motion (repeated three times), [5] repeating steps 2–4 ten times, [6] finishing with step 1. The self-massage techniques were adapted from the Academy of Breastfeeding Medicine Clinical Protocol [31] and previous studies [32, 33]. Education materials were also provided for participants to take home.

After the education session, participants were treated with five minutes of therapeutic ultrasound (pulsed mode) at a frequency of 1 MHz, a duty cycle of 20%, and a pulse intensity of 1.8 watts/cm^2^ [10, 34]. The ultrasound probe (probed size of 1 cm) was moved at a speed of about 4 cm/Sect. [34]. The intensity and duration were adjusted if the participant complained of discomfort. The ultrasound transducer head was massaged over the tender point on the breast [34]. As previous RCT reported no superior effect of a thermal (continuous) ultrasound over the sham treatment [15], we intended to maximize therapeutic effects by choosing a pulsed mode that was frequently used to decrease soft tissue swelling or inflammation [34, 35].

The therapeutic ultrasound session was followed by the physical therapist administered breast massage including general and focused massage. Participants were in the supine position. The breast massage was applied according to the Vodder method [36] to the affected breast and included the following steps [37]: (1) parallel rotary technique from sternum to axillae (repeated three times), (2) stationary circle (parallel and alternating) on the lateral side of the breast (repeated three times for each) (3) pump technique with one hand (from medical toward areola) and rotary-like motion with another hand (from areola toward axilla) (repeated three times), (4) alternating rotary technique over the ribcage below the breast (repeated three times), (5) small oval stationary circles over the intercostal spaces (repeated three times), (6) light pressure over the junction of the ribs at the sternum, (7) large stationary circles from waist to axilla (repeated three times), (8) repeat steps 2–7 three times, (9) parallel rotary strokes and parallel circles over the lower ribcage and below the breast (repeated three times), (10) final effleurage on the upper chest.

#### Sham group

Participants allocated to the ‘sham’ group received the same education and breast massage sessions as described above. In addition, the sham group received five minutes of ‘sham’ ultrasound at 0 W/cm^2^ intensity from a physical therapist. The participants did not know whether they were receiving real or sham ultrasound.

#### Usual care group

Participants allocated to the ‘Usual care’ group received usual obstetric care, which may include verbal advice/printed patient information regarding breast symptoms and breastfeeding from the medical or nursing staff.

### Data analysis

The data were analyzed using intention-to-treat principles with SPSS version 20.0 for Windows. The last observation carried forward imputation was used for imputing missing data. Descriptive statistics were used to summarize and report data. All outcome data were assessed for normality using the Kolmogorov-Smirnov test. If there were any significant differences in key variables between groups at baseline, these were used as co-variates for between-group comparisons. Outcome variable changes were analyzed with repeated measures analysis of variance using Bonferroni post hoc comparisons. Equivalent non-parametric tests were used if data were not normally distributed. Numbers, percentages, mean, standard deviation, mean difference, 95% confidence intervals, and *p*-values were reported. All analyses were tested with a significance level of p < 0.05.

## Results

**Fig. 1:**
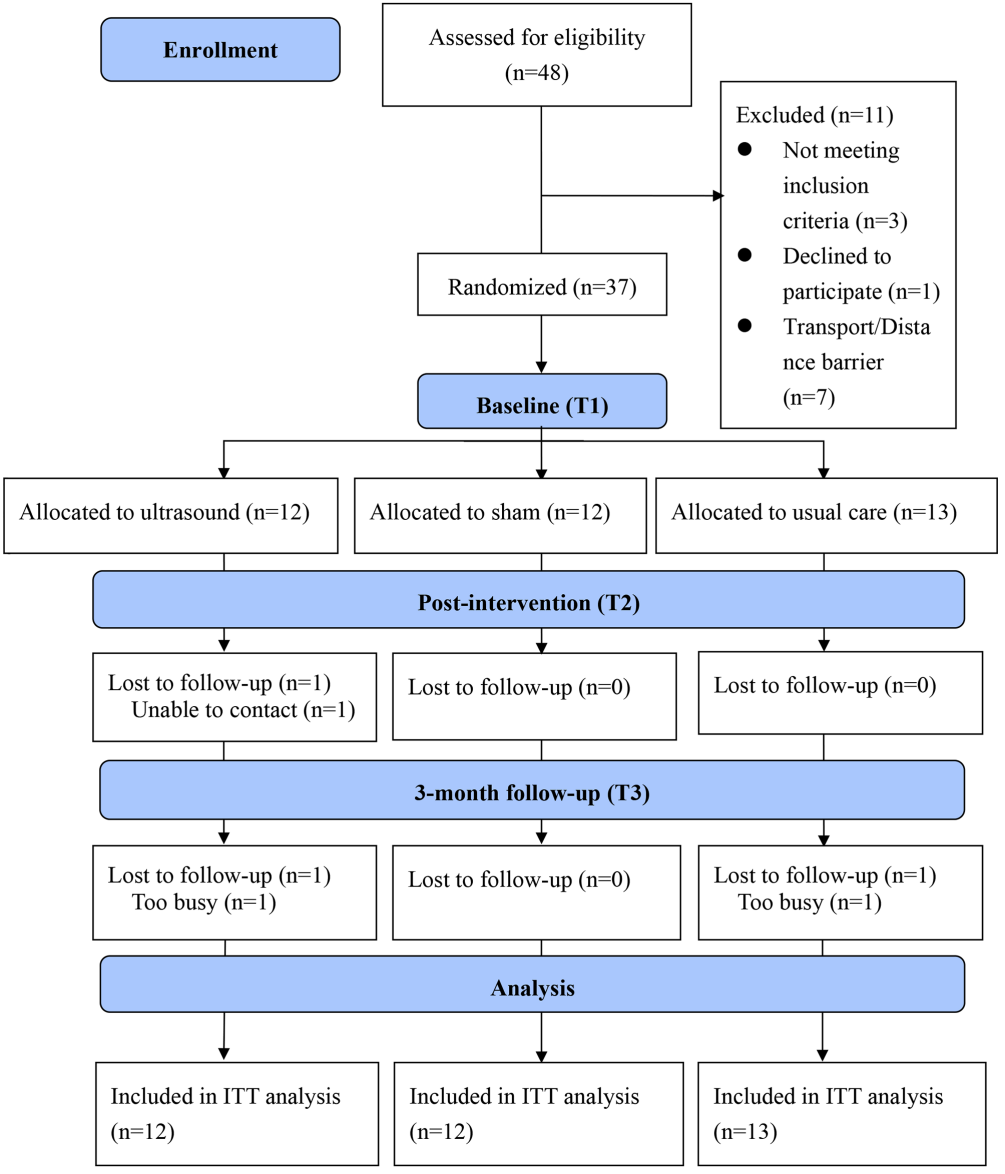
Flow of participants through the trial

A total of 37 participants were recruited and randomized to the ultrasound group (n = 12), sham group (n = 12), and usual care group (n = 13) (Fig. 1). One participant in the ultrasound group could not be contacted before completing the intervention, and two dropped out due to busy schedules at T3. No adverse events occurred during the study period. At T2, all participants in the ultrasound group (100%), 91.7% in the sham group, and 61.5% in the usual care group reported being very satisfied with the intervention, and the acceptability of the intervention was very high, with 100% of participants in the ultrasound group, 91.7% in the sham group, and 53.8% in a usual care group expressing high satisfaction.

The demographic characteristics of the participants are displayed in Table 1. The mean number of days after delivery was 25.8 (25.1), and 62.2% (n = 23/37) had a vaginal delivery. The most commonly reported breast symptoms were lump (94.6%) followed by pain (81.1%). There were no significant differences in participant characteristics between the three groups, except for the presence of a medical history.

**Table 1:** Participant characteristics

Variables	Ultrasound (n = 12)	Sham (n = 12)	Usual care (n = 13)
Age (year), mean ± SD	34.5 ± 3.3	33.6 ± 3.7	34.2 ± 4.4
BMI (kg/m2), mean ± SD	24.6 ± 3.4	24.1 ± 3.7	24.0 ± 3.7
Waist-hip ratio, mean ± SD	0.83 ± 0.04	0.82 ± 0.06	0.82 ± 0.04
Days after delivery, mean ± SD	16.8 ± 9.7	28.2 ± 34.8	31.9 ± 23.9
Married, n (%)	12 (100)	12 (100)	13 (100)
Living arrangement, n (%)			
With spouse	3 (25)	1 (8.3)	1 (7.7)
With child (including spouse)	9 (75)	11 (91.7)	12 (92.3)
Highest level of education, n (%)			
Master degree	2 (16.7)	5 (41.7)	5 (38.5)
Bachelor degree	9 (75.0)	7 (58.3)	7 (53.8)
Associate degree	1 (8.3)	0 (0)	0 (0)
Senior high school	0 (0)	0 (0)	1 (7.7)
Employment status, n (%)			
Full-time	9 (75.0)	9 (75.0)	10 (76.9)
Part-time	0 (0)	0 (0)	1 (7.7)
None	3 (25.0)	3 (25.0)	2 (15.4)
Smoking status, n (%)			
Never smoked	12 (100)	12 (100)	12 (92.3)
Ex-smoker	0 (0)	0 (0)	1 (7.7)
Medical history*, n (%)			
None^a^	8 (33.3)	8 (66.7)	12 (92.3)
Cold intolerance	1 (8.3)	0 (0)	0 (0)
Migraine	1 (8.3)	2 (16.7)	0 (0)
Dermatitis	1 (8.3)	0 (0)	0 (0)
Eczema^a^	4 (33.3)	0 (0)	0 (0)
Breast surgery	0 (0)	1 (8.3)	0 (0)
Ankyloglossia	0 (0)	0	1 (7.7)
Other	2 (16.7)	2 (16.7)	0 (0)
Medication, n (%)			
None	9 (75.0)	5 (41.7)	7 (53.8)
Chinese medicine	0 (0)	1 (8.3)	1 (7.7)
Lecithin	0 (0)	0 (0)	1 (7.7)
Butin	1 (8.3)	0 (0)	0 (0)
Antibiotics	1 (8.3)	1 (8.3)	2 (15.4)
Pain killer	2 (16.7)	3 (25.0)	3 (23.1)
Antihistamine	1 (8.3)	0 (0)	0 (0)
Stool softener	0 (0)	1 (8.3)	0 (0)
Anti-inflammatory	0 (0)	1 (8.3)	0 (0)
High blood pressure drug	0 (0)	1 (8.3)	0 (0)
Uterotonic drug	0 (0)	1 (8.3)	0 (0)
Gravidity, n (%)			
1	5 (41.7)	6 (50.0)	5 (38.5)
2	6 (50.0)	4 (33.3)	3 (23.1)
3	0 (0)	1 (8.3)	4 (30.8)
4	0 (0)	1 (8.3)	1 (7.7)
5	1 (8.3)	0 (0)	0 (0)
Parity, n (%)			
1	6 (50.0)	7 (58.3)	7 (53.8)
2	5 (41.7)	5 (41.7)	3 (23.1)
3	0 (0)	0 (0)	3 (23.1)
4	1 (8.3)	0 (0)	0 (0)
Number of children (including this birth), n (%)			
1	6 (50)	7 (58.3)	7 (53.8)
2	4 (33.3)	5 (41.7)	3 (23.1)
3	1 (8.3)	0 (0)	3 (23.1)
> 3	1 (8.3)	0 (0)	0 (0)
Delivery method of last childbirth, n (%)			
Vaginal	8 (66.7)	7 (58.3)	8 (61.5)
Cesarean	4 (33.3)	5 (41.7)	5 (38.5)
Current breast symptom*, n (%)			
None	0 (0)	0 (0)	0 (0)
Pain	11 (91.7)	10 (83.3)	9 (69.2)
Redness	4 (33.3)	1 (8.3)	2 (15.4)
Lump	12 (100)	12 (100)	11 (84.6)
Secretion of nipple	0 (0)	0 (0)	0 (0)
Warmth	1 (8.3)	1 (8.3)	2 (15.4)
White spot	1 (8.3)	0 (0)	0 (0)
Current systemic symptom*, n (%)			
None	8 (66.7)	10 (83.3)	12 (92.3)
Fever > 38.5 ˚C	0 (0)	0 (0)	0 (0)
Flu-like illness	1 (8.3)	0 (0)	0 (0)
General malaise	3 (25.0)	2 (16.7)	1 (7.7)
Other	0 (0)	0 (0)	0 (0)
History of breast symptoms in previous breastfeeding experience, n (%)	5 (41.7)	4 (33.3)	5 (38.5)
Current breastfeeding type, n (%)			
Breast milk directly from the breast	0 (0)	2 (16.7)	2 (15.4)
Pumped breast milk from a bottle	2 (16.7)	0 (0)	0 (0)
Breast milk along with formula	10 (83.3)	10 (83.3)	11 (84.6)
Breastfeeding or pumping frequency, n (%)			
Every 2 h	1 (8.3)	1 (8.3)	1 (7.7)
Every 3 h	3 (25.0)	2 (16.7)	4 (30.8)
Every 4 h	7 (58.3)	8 (66.7)	8 (61.5)
Every 5 h	0 (0)	1 (8.3)	0 (0)
other	1 (8.3)	0 (0)	0 (0)
Self-management strategy*, n (%)			
None	0 (0)	0 (0)	2 (15.4)
Correct latch-on techniques	3 (25.0)	3 (25.0)	4 (30.8)
Breastfeed the baby more frequently	3 (25.0)	3 (25.0)	7 (53.8)
Self-massage the breast	11 (91.7)	9 (75.0)	10 (76.9)
Frequently express milk by hand or with a pump	4 (33.3)	7 (58.3)	7 (53.8)
Relax and rest	6 (50.0)	5 (41.7)	7 (53.8)
Use a supplemental nursing device	6 (50.0)	5 (41.7)	7 (53.8)
Use warm/cold compresses	9 (75.0)	10 (83.3)	7 (53.8)
Other	0 (0)	2 (16.7)	1 (7.7)
Treatment received for breast symptoms before enrolment into this study*, n (%)			
None	10 (83.3)	11 (91.7)	12 (92.3)
Pain killer	1 (8.3)	1 (8.3)	1 (7.7)
Antibiotics	1 (8.3)	0 (0)	1 (7.7)
Antimycotics	0 (0)	0 (0)	0 (0)
Steroids	0 (0)	0 (0)	0 (0)
Herbal medicines	0 (0)	0 (0)	0 (0)
Lubricants	0 (0)	0 (0)	0 (0)
Other	1 (8.3)	0 (0)	0 (0)

^*^ Participants could select multiple responses

^a^ p < 0.05 between groups

Ph.D., Doctor of Philosophy; BMI, body mass index

The comparison of each outcome within and between groups at different assessment time-points is presented in Table 2. No significant differences were observed in any of the outcomes at baseline among the three groups. Significant improvements were observed in the severity of breast pain, breast lump, and general malaise as measured by the NRS. The severity of breast engorgement appeared to improve following the ultrasound intervention, and these effects on the severity of pain and engorgement were sustained at the 3-month follow-up in the ultrasound group. When comparing the 3-month follow-up to the baseline, significant changes were noted in the severity of pain, redness, lump, general malaise, breastfeeding self-efficacy, and the severity of engorgement in the ultrasound group.

**Table 2:** Comparison of outcome measures within and between groups

Variables	T1	T2	T3	Friedman test *p*-value	Kruskal-Wallis test *p*-value (T1)	Kruskal-Wallis test *p*-value (T2)	Kruskal-Wallis test *p*-value (T3)
Severity of breast pain (NRS)					0.311	0.126	0.016^e^
Ultrasound	6 (4,7)	1.5 (0, 4)	0 (0, 0.75)	< 0.001^a,b,c^			
Sham	6 (2, 8.75)	1.5 (0, 2)	0 (0, 0)	< 0.001^a,b,c^			
Usual care	4 (3, 6)	3 (1.5, 4)	1 (0, 2)	0.001^a,b,c^			
Severity of breast redness (NRS)					0.986	0.226	0.185
Ultrasound	0.50 (0, 4.0)	0 (0, 2.75)	0 (0,0)	0.011^c^			
Sham	0 (0, 5)	0 (0, 1)	0 (0, 0)	0.026^c^			
Usual care	1 (0, 3)	1 (0, 2)	0 (0, 0.5)	0.006^b,c^			
Severity of breast lump (NRS)					0.089	0.620	0.344
Ultrasound	7 (5.25, 8)	3.5 (1.5, 5)	0.5 (0, 4.5)	< 0.001^a,c^			
Sham	5 (2, 7.5)	2.5 (1.25, 5)	0 (0, 2)	0.001^a,b,c^			
Usual care	5 (3, 7)	4 (2.5, 5)	2 (0, 3.5)	0.004^b,c^			
Severity of general malaise (NRS)					0.158	0.506	0.779
Ultrasound	3.5 (0.25, 6.0)	0 (0, 1)	0 (0, 0)	< 0.001^a,c^			
Sham	3.5 (0, 5)	0.5 (0, 2)	0 (0, 0)	0.006^b,c^			
Usual care	1 (0, 3)	1 (0, 2)	0 (0, 0)	0.004^a,c^			
BESE total score					0.384	0.175	0.437
Ultrasound	40.0 (33.3, 49.0)	47.0 (31.75, 53.75)	52.0 (40.0, 58.0)	0.032^b,c^			
Sham	49.5 (39.25, 62.5)	56.5 (41.75, 66.0)	59.5 (34.25, 68.75)	0.018^a^			
Usual care	43 (37.5, 49)	46 (42, 51.5)	55 (52.5, 59.5)	< 0.001^a,b,c^			
Severity of breast engorgement					0.264	0.028^d,e^	0.310
Ultrasound	4.5 (4, 5)	2 (2, 3)	1 (1, 2)	< 0.001^a,b,c^			
Sham	4 (3.3, 4.8)	2 (2, 3)	1.5 (1, 2)	< 0.001^a,b,c^			
Usual care	4 (4, 4)	4 (2.5, 4.5)	2 (1, 3)	0.002^b,c^			
Breast hardness					0.361	0.508	0.100
Ultrasound	9.7 (4.0, 18.5)	4.0 (2.4, 14.5)	5.2 (2.8, 7.4)	0.323			
Sham	4.5 (1.7, 13.8)	4.3 (1.8, 13.4)	1.0 (0.2, 5.9)	0.098			
Usual care	10.8 (5.1, 19.7	7.7 (3.3, 20.2)	2.3 (1.1, 3.7)	0.003^b,c^			
Breast temperature (°C)					0.617	0.947	0.312
Ultrasound	37.1 (36.8, 37.4)	37.0 (36.7, 37.4)	36.9 (36.7, 37.3)	0.917			
Sham	37.2 (36.9, 37.6)	36.9 (36.7, 37.4)	36.7 (36.6, 37.1)	0.002^a,c^			
Usual care	37.6 (36.5, 38.2)	36.8 (36.6, 37.4)	37.0 (36.7, 37.4)	0.080			
Body temperature (°C)					0.116	0.089	0.035^d^
Ultrasound	36.5 (36.3, 36.5)	36.5 (36.3, 36.7)	36.4 (36.3, 36.5)	0.592			
Sham	36.6 (36.4, 36.9)	36.6 (36.4, 36.7)	36.4 (36.1, 36.7)	0.274			
Usual care	36.4 (36.3, 36.6)	36.3 (36.3, 36.6)	36.2 (36.0, 36.3)	0.132			
Volume of milk (g)					0.600	0.476	0.386
Ultrasound	26.1 (16.6, 61.0)	39.4 (19.3, 81.5)	52.2 (20.6, 81.5)	0.388			
Sham	22.5 (10.1, 32.8)	16.1 (5.1, 31.1)	17.8 (2.6, 35.3)	0.544			
Usual care	55.3 (17.1, 81.8)	62 (15.8, 79)	40.7 (16.9, 112.0)	0.775			

Data are median (IQR), unless stated otherwise

^a^ post-hoc analysis (Wilcoxon Signed Ranks Test), T2-T1, p < 0.05

^b^ post-hoc analysis (Wilcoxon Signed Ranks Test), T3-T2, p < 0.05

^c^ post-hoc analysis (Wilcoxon Signed Ranks Test), T3-T1, p < 0.05

^d^ post-hoc analysis (Mann-Whitney Test), Ultrasound vs. Usual care, p < 0.05

^e^ post-hoc analysis (Mann-Whitney Test), Sham vs. Usual care, p < 0.05

Significant differences were found in the severity of breast engorgement when comparing the ultrasound group and sham group to the usual care group at T2. A greater improvement in the severity of breast pain was observed in the sham group compared to the usual care group at T3. Additionally, a significant difference in body temperature was also observed between the ultrasound group and the usual care group at T3.

## Discussion

In this study, we found that participants with breast symptoms who received a physical therapy program consisting of therapeutic ultrasound, education, and massage demonstrated greater improvement in the severity of breast engorgement than the usual care group. However, there was no difference in improvements between the ultrasound and sham group. No adverse events were reported during the study period, and all participants in the ultrasound group reported being very satisfied with the intervention, considering the program highly acceptable for this population. This study suggests that physical therapy intervention is generally safe and beneficial for treating breast symptoms during lactation.

Therapeutic ultrasound, education, and massage are the most common interventions provided by physical therapists to mothers with breast symptoms in clinical practice [10]. Our findings support the use of these interventions as monotherapy or combination therapy for the alleviating breast symptoms after childbirth, as all three groups in our study showed significant improvements in self-reported severity of breast symptoms after interventions. Previous studies had also reported the effectiveness of breast massage alone [38], therapeutic ultrasound combined with conventional therapy including massage, hot moist pack, proper latching technique and/or proper fitting bra [14, 17], and breast massage combined with laser therapy, breast pumping, cold compress, and education [39] in relieving breast engorgement, breast pain, and breast lump. Therefore, physical therapy for lactating women with breast symptoms is essential and beneficial.

While our findings suggest that combinations of ultrasound, massage and education, and massage and education may be superior to usual care (i.e., education alone) in reducing the severity of breast engorgement, no additive effect of therapeutic ultrasound was found in treating breast symptoms and improving breastfeeding self-efficacy when compared to the sham group. These findings are in agreement with the study by Mclachlan et al. which reported improvements in subjectively perceived pain and hardness after both ultrasound and sham interventions in women with breast engorgement [15]. Although the low-power ultrasound with a frequency of 1 MHz may penetrate about 3–5 cm below the skin and induce effects through thermal and nonthermal mechanisms [40], the improvements found in both ultrasound and sham groups suggest that the ultrasound effects may be attributed to increasing cell wall permeability, micro-massage, and heat generated by the transducer, which helps to open the duct, increase circulation, improve the flow of human-milk, and reduce pain [15, 40]. Further, the lack of intergroup differences between the ultrasound group and sham group in all outcome measures may be related to the small sample size and the placebo effect [41].

While breast temperatures significantly improved in the sham group after intervention (36.9 °C at T2 and 36.7 °C at T3), the values remained above the average breast temperature (34.6 ± 0.7 °C) in healthy women [42]. Moreover, the body temperature was statistically and significantly different between the ultrasound group and the usual care group at T3. However, it should be noted that the mean temperature values in all three groups at each assessment time-point were within the normal range (36.1-37.2 °C) [43]. As the breast temperature in women is directly related to core body temperature and room temperature [42], future studies should consider measuring room temperature and correcting for room and core body temperature variations when recording breast temperature.

The discrepancies between significant improvements in the subjective measures of symptom severity and no significant findings in the objective assessments, such as breast hardness, breast and body temperatures, and volume of expressed human-milk, may be due to the impact of the placebo effect or the methods of objective measurement. Due to the lack of universally standardized measurement methods for breast hardness and breast temperature, we followed the methods used in previous studies [23, 24], and the locations where the measurements were taken may not be identical to the spots where participants had the symptoms. Future studies should utilize validated patient-reported outcome measures (e.g., Breast Inflammatory Symptom Severity Index [44, 45]) for assessing breast symptom severity and develop standardized and validated methods for assessing breast hardness and temperature.

As the therapeutic ultrasound (1 MHz, 1.8 watts/cm^2^, pulsed mode for five minutes) was not superior to sham ultrasound when added to education and breast massage in the treatment of breast symptoms in this study, the inclusion of therapeutic ultrasound is not recommended as a mandatory component in the multimodal physical therapy treatment of breast symptoms in lactating women. Our findings align with the conclusions of previous reviews [11, 38, 46] that ultrasound is not effective in improving breast symptoms in breastfeeding women, and the improvement in symptoms may be attributed to the placebo effect, impacts of breast massage, or the spontaneous alleviation of symptoms as women continue to breastfeed. Future trials could include a fourth group receiving breast massages and education only to provide evidence on the effects of different combinations of physical therapy modalities and to investigate the most cost-effective physical therapy program for this population.

### Limitations

Our study explored the short-term and medium-term effects of physical therapy interventions for lactating women with breast symptoms and may add valuable information to the evidence base. However, the small sample size was the major limitation of this study due to slow recruitment, which was severely impacted by visiting restrictions during the COVID-19 pandemic; therefore, our results should be interpreted with caution. Despite the slow recruitment rate, high satisfaction and acceptability with both ultrasound and sham ultrasound interventions confirm the feasibility of the physical therapy program in this cohort. The ultrasound parameters used in our study were adapted from previous studies [10, 34]; nevertheless, the dosage and treatment duration may be insufficient to have an impact on the objective outcomes, and the assessment methods may not be optimal for this population. Further investigation into the specific ultrasound dosage and the measurement methods for breast hardness and temperature in women with breast symptoms needs to be performed in larger trials.

## Conclusion

Physical therapy intervention, including therapeutic ultrasound, education and massage, and sham ultrasound, may be beneficial and acceptable to lactating women with breast symptoms. However, ultrasound did not have an additional effect when compared to the sham ultrasound. The combined physical therapy modalities may be considered as an adjunct to monotherapy for lactating women with breast symptoms. Larger randomized controlled trials are needed to confirm the findings of this study.

## Data Availability

The datasets generated and/or analyzed during the current study are not publicly available due to the need to preserve respondent confidentiality but are available from the corresponding author upon reasonable request.

## Abbreviations

BSES-SF: Breastfeeding Self-Efficacy Scale – Short Form
CCLA: Chinese Certified Lactation Assistants
IBCLC: International Board Certified Lactation Consultants
IRB: Institutional Review Board
NRS: numerical rating scale
RCT: Randomized controlled trial
T1: Baseline
T2: Immediately post-intervention
T3: 3 months following baseline
